# Qualitative investigation of factors impacting pre‐exposure prophylaxis initiation and adherence in sexual minority men

**DOI:** 10.1111/hex.13382

**Published:** 2021-12-14

**Authors:** Marcus Alt, Paul Rotert, Kate Conover, Sarah Dashwood, Andrew T. Schramm

**Affiliations:** ^1^ Department of Psychiatry & Behavioral Sciences University of Kansas Medical Center Westwood Kansas USA; ^2^ Department of Family Medicine & Community Health University of Kansas Medical Center Westwood Kansas USA; ^3^ Department of Surgery Division of Trauma & Acute Care Surgery, Medical College of Wisconsin Milwaukee Wisconsin USA

**Keywords:** HIV prevention, PrEP, sexual minority men

## Abstract

**Introduction:**

Men who have sex with men continue to account for the majority of new HIV infections in the United States. Many of those with new infections are unaware that they have HIV. Preventative measures continue to be essential in reducing new infections, with pre‐exposure prophylaxis (PrEP) being widely recommended.

**Objectives:**

The overall aim of this qualitative study is to explore the impact of stigma, patient–provider dynamics and patient perception of PrEP on men's engagement with PrEP in a primary care setting.

**Methods:**

The Consensual Qualitative Research Methodology (Hill, 2012) was used to explore the experiences of 14 men receiving care for PrEP at a Family Medicine clinic in the Midwest. Semistructured interviews were conducted to allow for depth of understanding of individuals' experience.

**Results:**

Four major domains were identified: motivation to pursue PrEP, barriers and adherence to care, beliefs about how PrEP is perceived by others and experiences discussing sexual health and PrEP with providers.

**Conclusion:**

It is important to better understand factors contributing to the pursuit of and adherence to HIV prevention measures and HIV care. Further, health systems and providers are encouraged to consider opportunities in terms of how their practice can destigmatize PrEP use and offer a welcoming environment for those pursuing HIV prevention.

**Patient or Public Contribution:**

Patients were involved in the study through their participation in semistructured interviews, which provided the data analysed for this study. There was no additional participation beyond the one‐time interview or follow‐up poststudy. Their interviews helped contribute to our better understanding of the needs and experiences of those receiving PrEP‐related care.

## 1 INTRODUCTION

Despite great gains in efforts to prevent HIV,^1^ as of 2018, an estimated 1.17 million individuals aged ≥13 years in the United States were living with the virus.^2^ In 2018, 37,968 individuals in the United States were newly diagnosed with HIV, and 69% of those newly diagnosed were gay or bisexual men.^1^ These figures illustrate that the burden of HIV is not equally distributed.^3^ Despite overall declines in HIV, the rates of new diagnoses continue to increase among men who have sex with men (MSM).^4^ Although only 7% of men report having sexual contact with other men, 82.9% of all HIV infections among men were attributed to male‐to‐male sexual contact. The majority of new HIV transmissions are from persons who do not know they have HIV, highlighting the importance of prophylactic prevention measures.^5^

The introduction of pre‐exposure prophylaxis (PrEP)^6^ for HIV prevention in 2012 transformed the fight against the global HIV/AIDS pandemic.^7^ For the first time, individuals who were HIV‐negative could take a well‐tolerated pill once daily and significantly reduce their likelihood of seroconversion if exposed to the virus.^8^, ^9^ Best practices now include recommending PrEP for individuals at high risk for acquiring HIV due to sexual practices or intravenous substance use. PrEP is recommended by the CDC and received a Grade A recommendation from the US Preventive Services Task Force (2019).^10^

Despite the great promise of PrEP, uptake has been much slower than expected.^6^, ^11^ Only 18.1% of the estimated 1.2 million persons in the United States for whom PrEP is recommended are prescribed a PrEP medication.^12^ Although MSM, in comparison to other at‐risk populations, have a higher rate of uptake, the public health benefit of PrEP has been limited by relatively few individuals taking it.^6^, ^13^

Another challenge is PrEP patients' adherence to medication use as prescribed, which varies widely from 22% to 90%.^14^ Because the public health benefits of PrEP are dependent on taking the medication daily,^6^ factors impacting PrEP adherence are an important target for research.^15^ To fully realize the potential public health benefits of PrEP, barriers to initiation and adherence must be better understood. This information is needed to inform subsequent interventions to improve uptake and effective use.

Individuals with a stigmatized identity (e.g., being LGBTQ) face unique stressors related to that identity, such as experiences with discrimination, efforts to conceal one's identity and the internalization of negative messages about their identity.^16^ These stressors, which represent a concept called minority stress,^17^ have powerful impacts on behaviour,^18^ including engagement in health care services, and drive health disparities within LGBTQ communities.^19^, ^20^, ^21^ Uptake of PrEP for HIV prevention is hindered by minority stress, particularly within target populations.^22^, ^23^ One qualitative study found that LGBTQ and MSM participants feared rejection from partners and being labelled as sexually promiscuous as a result of using PrEP.^24^ One manifestation of minority stress is the internalization of stigma.^25^ For sexual minority individuals, this can be conceptualized as internalized homophobia.^17^ Internalized homophobia has been shown to negatively impact PrEP use,^26^, ^27^ suggesting that multiple manifestations of stigma impact PrEP use.

Another important factor to consider is the practice of health care providers. Increased utilization is associated with effective education and messaging about PrEP.^28^ However, coverage of PrEP in medical education is highly variable,^29^ and providers' medical decision‐making related to PrEP is affected by heterosexism and racism.^30^ Disturbingly, medical students are less likely to prescribe PrEP for patients at highest risk for seroconversion (i.e., less often prescribing it for patients who do not use condoms and have multiple partners).^31^ Provider notions that prescribing PrEP may lead to risk compensation (i.e., patients taking greater risks with sexual health once they are prescribed PrEP) also represent a provider‐level factor that has interfered with more widespread PrEP use.^32^ These issues have led to the addition and refinement of PrEP‐specific training as part of medical education.^29^ Although these studies suggest that stigma and provider attitudes and behaviour have an important influence on PrEP usage, previous research (e.g., 28‐32) has focused on provider perspectives on the phenomena. As described in detail below, we sought to shed light on the patient experiences of these phenomena. Evaluation of patients' perceptions of provider medical decision‐making and approach to patient communication surrounding PrEP is a poorly understood but potentially important part of increasing uptake of PrEP.

### 1.1 Present study

To elucidate the role of stigma and provider behaviour in individuals' PrEP initiation and adherence, we conducted a qualitative study using semistructured interviews. Participants were patients already on PrEP and recruited from a Family Medicine clinic at an academic medical centre in the Midwest urban setting. We sought to explore the perception of PrEP use among MSM and its impact on one's decision to pursue PrEP, the impact of internalized homonegativity or stigma of PrEP use and aimed to gain a better understanding of the experience of communicating with providers about sexual health in relation to HIV prevention.

## 2 METHODS

Fifteen men (*N* = 15) receiving medical care for PrEP in a Family Medicine clinic were recruited for participation. One individual did not follow up with participation after initial contact; therefore, the sample size was 14 self‐identified cisgender gay or bisexual men (*N* = 14). A qualitative methodological approach was utilized to allow for a depth of understanding into individuals' experiences. Generally, qualitative research allows for an understanding into the complex factors contributing to a particular construct(s). This is often gained through individual interviews, as is the case with the current study.

This sample size is consistent with the CQR Methodology as outlined by Hill (2012), which recommends including 12–15 participants. Within CQR, this sample size generally allows for consistency in response, given a relatively homogeneous sample, which is appropriate for this study. Table 1 presents a description of participants' relevant demographic information including age, race/ethnicity, sexual orientation, HIV status and relationship status.

**Table 1:** Demographics

Participant	Age	Race/ethnicity	Sexual orientation	Relationship status
1	27	White/Caucasian	Gay	Single
2	57	Black/African American	Gay	Single
3	27	White/Caucasian	Gay	Partnered
4	42	Hispanic and Latino	Gay	Partnered
5	55	White/Caucasian	Gay	Single
6	42	White/Caucasian	Gay	Single
7	26	Black/African American	Bisexual	Single
8	37	White/Caucasian	Bisexual	Single
9	53	Hispanic and Latino	Gay	Partnered
10	28	White/Caucasian	Gay	Single
11	23	Black/African American	Gay	Single
12	32	White/Caucasian	Gay	Single
13	27	White/Caucasian	Gay	Single
14	23	White/Caucasian	Gay	Single

*Note*: This table includes demographic information as self‐reported by participants. All participants endorsed HIV status as negative, and thus not included in this table.

The study was subjected to IRB review, with approval. Before the medical appointment, the clinical nurse coordinator identified eligible patients. Initial criteria included patients who were currently receiving medical care in the clinic for PrEP. If identified by the nurse coordinator, information on participation in the study was presented by the nurse coordinator during the medical appointment. After reviewing a consent form, eligible patients who opted to participate provided written consent. Participants were informed that their participation would not impact their medical care. Signed consent forms, including preferred contact method, were then provided to the principal investigator (PI), who initiated scheduling. The PI then scheduled the interview with consenting participants. No financial compensation was provided.

Participants took part in a semistructured interview with the PI. The interview took place on a private phone line in a private room where confidentiality could be maintained and was recorded using a digital recorder with participants' consent. The PI reviewed the consent form with the participant and allowed any questions or clarifications. The purpose of the study was summarized, and demographic information, including sexual orientation, was reviewed to ensure eligibility. Participants could stop the interview at any time, and the PI checked in on any levels of distress during the interview. Recorded interviews were transcribed verbatim by the research team.

### 2.1 Consensual Qualitative Research (CQR) Methodology

Analysis of the data closely followed the CQR Methodology, as described by Hill.^33^ Transcriptions were first reviewed to identify broad topic areas to help establish the domains. The PI completed an initial review of transcripts. All transcripts were then reviewed by team members, and data were ‘blocked’ into relevant domains^33^ until consensus was achieved. The established domain list was reviewed by the external auditor, with relevant changes made accordingly.

A similar review process took place in the summarization of domains into core ideas (workable descriptions of the data) as well as categories/subcategories (common themes across the interviews and that help define the content of the domains and core ideas). Finally, the frequency or representativeness of the categories/subcategories was determined. Categories/subcategories were labelled as general, typical or variant. General indicates categories in all or all but one of the interviews. Typical is found in more than half and up to all but one of the interviews. Variant categories are found to be in at least two and up to half of the interviews. No frequency label was assigned to any categories/subcategories that were found in less than two of the interviews.

## 3 RESULTS

No participant expressed distress during the interviews. Four primary domains emerged from the data: Pursuit of PrEP; Adherence and Care; Perception and Stigma; and Interaction with Medical Providers. Table 2 presents an organized overview of the data. The following section offers a description of each of the domains and narrative examples illustrating the categories/subcategories that arose from the data.

**Table 2:** Domains, categories, subcategories and frequency

Domain/category/subcategory	Frequency
1. Pursuit of PrEP	
a. Motivation for use: HIV prevention	General
b. Information source	
i. Media	Typical
ii. Social contact	Typical
iii. Medical team	Variant
c. Length of use	
i. Less than a year	Variant
ii. More than a year	Typical
2. Adherence and Care	
a. No difficulty adhering	Typical
b. Issues with clinic appointments	
i. No barriers	Typical
ii. Scheduling difficulty	Variant
iii. Communication access to provider and support staff	Variant
c. Challenges with insurance and financial resources	Typical
3. Perception and Stigma	
a. Social perception of PrEP use	
i. PrEP allows for safer sex by decreasing HIV risk	Variant
ii. PrEP use seen as excuse for risky sexual behaviour	Typical
b. Disclosure of PrEP use	
i. Fully open with others	Variant
ii. Not open with others	Variant
iii. Open to some	Variant
c. Social stigma experienced	
i. No negative stigma	Variant
ii. Some stigma around use and high‐risk behaviour	Variant
iii. Assumption PrEP is only for gay men	Variant
d. No influence of stigma on PrEP use	Typical
e. Past experiences of internalized homophobia	Variant
f. Internalized homophobia and initiating PrEP	
i. Not associated with decision to pursue PrEP	Typical
ii. Partial factor in decision to pursue PrEP	Variant
iii. Significant factor in decision to pursue PrEP	Variant
g. PrEP use encouraged in social groups	Variant
4. Interaction with Medical Providers	
a. Feelings on sharing personal information with medical team	
i. Uncomfortable but willing to disclose for appropriate medical care	Typical
ii. Comfortable and willing to disclose for appropriate medical care	Variant
iii. Not fully open about sexual practice	Variant
b. Important to share sexual and gender identity, not sexual preferences	Variant
c. Things that impact comfort level with providers	
i. Nothing would make it less comfortable	Variant
ii. Paper forms	Variant
iii. Acceptance and approachability	Typical
d. Initiating conversations on sexual health	
i. Better for physicians to initiate discussion	Variant
ii. More information about PrEP needed	Variant
iii. Patient often initiates discussion	Variant
e. Medical providers need growth in providing care to LGBTQ patients	Typical

Abbreviation: PrEP, pre‐exposure prophylaxis.

### 3.1 Pursuit of PrEP

The Pursuit of PrEP domain focused on motivation to initiate PrEP. Participants described having good knowledge of PrEP as a form of HIV prevention before starting. One participant stated:

To be honest, the reason that I wanted to pursue PrEP is because I don't want HIV. I know that me, being a gay guy and having sex with men, puts me at higher risk for HIV.

Messaging of PrEP use appeared to be prevalent in multiple sources, including media (i.e., internet blogs and TV commercials):

I think when I saw it on Facebook from one of those gay news outlets. They were covering a story on a new drug that it definitely helps you not get HIV. So that was definitely a selling point.

Within social contacts and peer groups:

Yeah and I was also like just kind of playing volleyball that was a lot of med students that talked about it and so it just kind of sparked my interest.

As well as from their medical team directly:

A doctor brought it up. We were having discussions at my annual physical…I talked about that I am bi, so he suggested that this would be a good thing to be on because it is a preventative measure more than anything else.

### 3.2 Adherence and Care

Participants discussed the barriers associated with adherence to PrEP. They described few obstacles with maintaining the required daily dosage:

I haven't really had any trouble sticking to it. It has become a part of my morning routine.

Further, participants described ease of access to the medical team to help address any concerns around prescriptions and appointments:

I have felt that there has been very easy and quick access to physician staff. I use the web portal to leave messages for (doctor) or his staff.

Despite these, several participants described financial and insurance issues. Due to inconsistencies in coverage for PrEP, alternative financial support became the only option for some:

Like if you don't apply for at least the assistance or get the co‐pay card through Gilead. I don't know how people afford it.

### 3.3 Perception and Stigma

Participants were also asked about how PrEP use is seen within and outside of the LGBTQ community. Broadly, participants addressed topics related to the perception of PrEP use and also how experiences of internalized homophobia and related stigma may impact one's decision to pursue PrEP. Individuals stated that some see PrEP as ‘permission’ to engage in risky sexual behaviour and misunderstand it as being protection from all STIs. For example:

Amongst my more mature friends—it is perceived as a great way to add a layer of protection during sex my more immature friends take it as a carte blanche. Some people do think that it protects you from everything, which I think is obviously stupid and incorrect.

Additionally, some described stigma that those who take PrEP engage in risky sexual behaviour:

I feel like there may be some kind of small stigma for people to use it mainly because I feel like there are people who view it as just a way for people to have condomless sex and that is not why I take it at least.

Participants described PrEP being seen as a ‘gay’ drug, even though it is beneficial for all. This leads to an opportunity for providers to engage in more open conversation and education of HIV prevention for all patient populations. For example:

It almost feels like if you are on PrEP then it is a gay stigma, but it is just important for heterosexuals to be on it. I think that is the stigma that is on it, like ‘I don't have to be on it because I'm straight versus gay.’

Despite this, PrEP was widely encouraged within their own social circles. Participants described it as being the ‘standard’ and expectation in the MSM community:

It's basically a standard. Like if you want to engage in sexual activity, you need to have PrEP if you are not then you are a higher risk person to have sex with. It's like, ‘Are you on PrEP or not? Yes? Okay good.’

Lastly, although some participants described experiencing internalized homophobia during their life, they generally did not feel that it impacted their decision to pursue PrEP.

I wouldn't really say that the negative experiences that I have had, hasn't led me to take PrEP. I felt like I just needed to do it…and I wanna protect myself.

There was acknowledgement of the potential impact of internalized homophobia on PrEP use, despite it not being a factor in their own decision:

I think it could be a barrier for some people even if it wasn't for me. It's like saying that if I am on PrEP, then I am confirming that I am gay or bisexual. I think part of the identity process is coming to terms with who you are is different than taking a prescription med.

### 3.4 Interactions with Medical Providers

The final domain, Interactions with Medical Providers, centered around discussion of sexual orientation, sexual activity and other relevant information with medical providers. There was also focus on how providers can be more effective in their care. Participants described discomfort in having conversations with medical providers about their sexual activity. Some specifically spoke about being asked about their sexual role preference and related discomfort:

I was nervous to tell him my sexual orientation and tell him what I like. He is asking me, ‘Are you the giver or the receiver?' I was embarrassed to answer those questions.

Despite the discomfort and personal nature of these questions, the importance of being open and honest with their providers to gain the best care was emphasized:

There (are) risks with certain sexual behaviors. I believe that doctors need to know about that so they can first educate the person to the possibilities, the risks, and let us know what we can do to minimize those risks. Especially with what we can get as a gay guy.

Some participants found it difficult to find a doctor whom they felt understood the unique needs of LGBTQ individuals and HIV prevention. This impacted their comfort level in opening up. Participants emphasized the importance of acknowledging sexuality across cultures to help with patient comfort level:

You know, if you go to someone that feels LGBTQ friendly or someone who has specifically worked with the PrEP program, I would feel very comfortable. But if I have someone who I don't know Who doesn't really stand out as being LGBTQ friendly I don't feel as comfortable.

Lastly, expression of support for the LGBTQ community would help some participants feel more comfortable in clinic:

I think it would be important to have your medical staff, not necessarily identify as an ally for an LGBT community, and not even asking that they feel forced to. But if there are any members—maybe like a pin or something or on their nametag.

## 4 DISCUSSION

PrEP has dramatically changed how we prevent HIV and is a key pillar in our fight to end the HIV epidemic.^2^ In this qualitative analysis of sexual minority men, we aimed to investigate the factors impacting PrEP initiation, adherence, perceptions and stigma and interaction with medical providers within a primary care clinic. Given the stigma associated with sexual health and HIV, understanding these factors is vital to ensuring that patients have a positive experience that encourages them to continue engagement in care over time. The goal of our study was to better understand the experiences of those receiving PrEP and communicate that information to medical providers so that they can provide high‐quality PrEP care.

Many participants stated that they were aware and motivated to start PrEP even before their clinic appointment. They initiated PrEP through a variety of methods including independently seeking PrEP care, being referred by a peer or being counselled by their physician to begin PrEP.

Participants described valuing the PrEP‐related experiences of their social groups. They reported a friend recommending PrEP, or having a good experience with PrEP care, as a major motivator for PrEP initiation. This encourages providers to ask their PrEP patients to share their clinic information within social networks as a method to attract new PrEP patients. All PrEP prescribing clinics and providers would benefit from registering themselves on the PrEP Locator website so that they can be easily found by patients (https://preplocator.org).

Participants also reported being more likely to start PrEP if recommended directly by their doctor. This highlights that it is important that all primary care providers learn about PrEP care, understand indications for initiation, how to prescribe PrEP and strongly recommend it to patients, when indicated. Lastly, patients who arrive at their appointment with existing knowledge of HIV risk and PrEP were more likely to initiate PrEP. This lends support to public health efforts to provide education on HIV risk and PrEP.

Fortunately, participants had little difficulty with adherence and accessing care. This was enabled by access to physicians with appropriate PrEP training in our research study setting and availability of direct communication with the medical team through an electronic medical record. One physician in this practice championed PrEP efforts beginning in 2017 and conducted regular PrEP training and education for fellow providers and clinic staff. A ‘PrEP Quick Reference Guide’ (see Supporting Information Appendix 1) was also created, regularly updated by the physician champion, and laminated at multiple locations in the clinic for easy reference. Other clinics aiming to increase PrEP prescribing would benefit from nominating a PrEP champion within their practice to help lead efforts and stay up to date with PrEP guidelines.

In addition to a physician champion, the clinic designated a nurse within the clinic to serve as the PrEP Nurse Care Coordinator. This individual was the initial point person for all nursing staff and patient questions. She completed Prior Authorizations and payment assistance programmes requests, removing this time‐consuming burden from the prescriber. She also built and maintained a PrEP patient database, contacted patients when they had missed appointments, and developed a follow‐up programme where patients saw the physician every 6 months and completed PrEP labs on the alternating 3 months without an appointment. Without a designated nurse who was knowledgeable about PrEP, many study participants expressed that they would have likely not continued the medication due to barriers with insurance, cost and the need for regular lab monitoring and follow‐up. Pharmacy colleagues, when available, can also serve as a crucial PrEP team member to assist with these barriers.

Stigma played a role in the initiation of PrEP, with some study participants commenting that it was thought of as a ‘gay’ drug. This points to the importance of educating providers and patients that PrEP is indicated for anyone at risk of HIV, regardless of their sexual orientation. Some participants believed that PrEP use would make others assume that they participated in riskier sexual behaviours, encouraging the medical community to destigmatize and normalize sexual health care, especially as it relates to PrEP. Others commented that friends viewed PrEP as universal protection against all sexually transmitted infections (STIs), emphasizing the importance of prescribers clearly stating that PrEP only protects against HIV and continued condom use is an important strategy to protect against HIV and other STIs.

Internalized homophobia and minority stress were also evaluated as potential barriers to PrEP initiation. Several participants noted previous experiences with internalized homophobia and recognized how this might be a barrier to initiation of PrEP, but generally did not view this as a personal barrier.

Participants reported discomfort in discussing sexual health with medical providers. Some participants were afraid that they would be judged and felt embarrassed discussing these topics in a medical setting. This discomfort was lessened by seeing the same provider on a regular basis and developing a relationship with that person. The continuity of care and longitudinal relationships developed within a primary care clinic serve as the ideal setting to discuss sexual health and PrEP.

Participants also expressed a desire for their provider to more openly state that they were ‘LGBTQ friendly’. Suggestions from participants included providers directly expressing this to patients while discussing PrEP, by wearing LBGTQ pins on name tags, or LGBTQ‐supportive signage. Clinics are encouraged to have staff undergo Safe Zone (https://thesafezoneproject.com/) or other similar training programmes to help educate all staff about how to best care for LGBTQ patients. More broadly, this study encourages medical schools and residency programmes to conduct more training on sexual health, LGBTQ care, stigma and PrEP. This includes early education about the benefits of PrEP, how to prescribe PrEP and how to take a sexual history for the LGBTQ community in a patient‐centred manner.

There are many previous studies that have used qualitative analysis to better understand PrEP care. A recent meta‐synthesis on qualitative PrEP research among MSM highlights six of the highest‐quality studies published between 2010 and 2018.^34^ Our methodology was similar to these studies; however, none were conducted in the Midwest and only one was conducted within a primary care clinic. Our research helps provide more qualitative PrEP data for the Midwest region of the United States and patient experiences within a primary care clinic.

In addition, our study is unique because it was conducted while the primary care clinic was working to build a new PrEP programme. There is a lack of qualitative data highlighting patient perspectives on new PrEP programmes within primary care clinics. Given that primary care clinics are the first point of contact for patients interacting with the health care system, a better understanding of these perspectives is key to successful implementation of PrEP programmes.

The PrEP Nurse Care Coordinator in this study is also an important contributor to the literature. Other previous research has evaluated nurse‐led PrEP initiatives, but the duties of the PrEP Nurse Care Coordinator in our study setting are unique.^35^ Barriers to PrEP initiation, adherence and follow‐up are well documented.^36^ Our patients noted few barriers in their care as a result of there being a dedicated PrEP programme with a PrEP Nurse Care Coordinator. Other clinics looking to start a new or improve an existing PrEP programme can benefit from these lessons learned on how to provide high‐quality, patient‐centred PrEP care.

### 4.1 Limitations & future directions

All subjects in this study received care from the same provider in a single clinic setting, potentially limiting the generalization of these results to all patients. However, this does provide greater consistency of the data to evaluate the impact of specific programmes, like the PrEP Nurse Care Coordinator, on patient care. The study intentionally included only sexually minority men; however, the majority of participants were non‐Hispanic, White and all had insurance. This may limit the applicability of the research to other genders, sexual orientations, races and those without insurance.

Future research could be conducted using a similar methodology at a clinic without a designated PrEP programme and PrEP Nurse Care Coordinator to evaluate the impact of these factors on care. Given the importance of perception, stigma and interaction with medical providers, a similar qualitative study could also focus on the perspective of medical providers as they progress through their training. Questions include level of comfort taking a sexual history, views on PrEP, previous sexual health and PrEP training, and comfort prescribing PrEP.

## 5 CONCLUSION

Understanding factors that impact initiation and adherence is key to ensure that patients get started on the medication, when indicated, and continue therapy while their risk of HIV is still present. A better understanding of these factors, as learned from this qualitative study, has the potential to improve the quality of PrEP care provided in primary care clinics.

## CONFLICT OF INTEREST

None of the authors of the above manuscript has declared any conflict of interest which may arise from being named as an author on the manuscript.

## AUTHOR CONTRIBUTIONS

Author Marcus Alt was responsible for the interviews, transcription of data, data review and final drafts of this manuscript. Marcus Alt and Paul Rotert were responsible for the development of this project, and the recruitment took place in collaboration with the nursing team in PR's practice. Kate Conover, Sarah Dashwood and Andrew T. Schramm were responsible for serving as internal and external auditors in data review, as well as manuscript development. All authors actively participated in editing drafts before submission.

## Supporting information

Supporting information.

## Data Availability

The data that support the findings of this study are available on request from the corresponding author. The data are not publicly available as they include information that could compromise the privacy of the participants.

## References

[1] Centers for Disease Control and Prevention Preexposure prophylaxis for the prevention of HIV infection in the United States—2018 update: a clinical practice guideline. 2018. Accessed February 01, 2021. https://www.cdc.gov/hiv/pdf/risk/prep/cdc-hiv-prep-guidelines-2017.pdf

[2] Centers for Disease Control and Prevention Estimated HIV incidence and prevalence in the United States, 2014‐2018. HIV Surveillance Supplemental Report 2020. 2020. Accessed February 01, 2021. http://www.cdc.gov/hiv/library/reports/hiv-surveillance.html

[3] Hess KL , Hu X , Lansky A , Mermin J , Hall HI . Lifetime risk of a diagnosis of HIV infection in the United States. Ann Epidemiol. 2017;27(4):238‐243. 28325538 10.1016/j.annepidem.2017.02.003 PMC5524204

[4] World Health Organization Consolidated guidelines on HIV prevention, diagnosis, treatment, and care for key populations: 2016 update. 2016. Accessed February 01, 2021. https://apps.who.int/iris/bitstream/handle/10665/246200/9789241511124-eng.pdf 27559558

[5] Li Z , Purcell DW , Sansom SL , Hayes D , Hall HI . Vital signs: HIV transmission along the continuum of care—United States, 2016. MMWR Morb Mortal Wkly Rep. 2019;68(11):267‐272. 30897075 10.15585/mmwr.mm6811e1 PMC6478059

[6] Grant RM , Anderson PL , McMahan V , et al. Uptake of pre‐exposure prophylaxis, sexual practices, and HIV incidence in men and transgender women who have sex with men: a cohort study. Lancet Infect Dis. 2014;14(9):820‐829. 25065857 10.1016/S1473-3099(14)70847-3 PMC6107918

[7] Holmes D . World Report: FDA paves the way for pre‐exposure HIV prophylaxis. Lancet. 2012;380:324‐325. 10.1016/s0140-6736(12)61235-5 22852138

[8] Fonner VA , Dalglish SL , Kennedy CE , et al. Effectiveness and safety of oral HIV preexposure prophylaxis for all populations. AIDS. 2016;30(12):1973‐1983. 27149090 10.1097/QAD.0000000000001145 PMC4949005

[9] Grohskopf LA , Chillag KL , Gvetadze R , et al. Randomized trial of clinical safety of daily oral tenofovir disoproxil fumarate among HIV‐uninfected men who have sex with men in the United States. J Acquir Immune Defic Syndr. 2013;64(1):79‐86. 23466649 10.1097/QAI.0b013e31828ece33

[10] US Preventive Services Task Force . Preexposure prophylaxis for the prevention of HIV infection: US Preventive Services Task Force Statement. JAMA. 2019;321:2203‐2213. 31184747 10.1001/jama.2019.6390

[11] Kaur KK , Allahbadia G , Singh M . Exploring the causes of preexposure prophylaxis of HIV failure—future developments for overcoming the same: a mini‐review. J Hum Virol Retrovirol. 2019;7:1‐5.

[12] Harris NS , Johnson AS , Huang YLA , et al. Vital signs: status of human immunodeficiency virus testing, viral suppression, and HIV preexposure prophylaxis—United States, 2013–2018. Morb Mortal Wkly Rep. 2019;68(48):1117‐1123. 10.15585/mmwr.mm6848e1 PMC6897528 31805031

[13] Krakower DS , Mimiaga MJ , Rosenberger JG , et al. Limited awareness and low immediate uptake of pre‐exposure prophylaxis among men who have sex with men using an internet social networking site. PLoS One. 2012;7(3):e33119. 22470438 10.1371/journal.pone.0033119 PMC3314648

[14] Chou R , Evans C , Hoverman A , et al. Preexposure prophylaxis for the prevention of HIV infection: evidence report and systematic review for the US Preventive Services Task Force. JAMA. 2019;321(22):2214‐2230. 31184746 10.1001/jama.2019.2591

[15] Stankevitz K , Grant H , Lloyd J , et al. Oral preexposure prophylaxis continuation, measurement and reporting. AIDS. 2020;34(12):1801‐1811. 32558660 10.1097/QAD.0000000000002598 PMC8635251

[16] Badenes‐Ribera L , Sanchez‐Meca J , Longobardi C . The relationship between internalized homophobia and intimate partner violence in same‐sex relationships: a meta‐analysis. Trauma Violence Abuse. 2017;20:13‐343. 10.1177/1524838017708781 29333955

[17] Meyer IH . Prejudice, social stress, and mental health in lesbian, gay, and bisexual populations: conceptual issues and research evidence. Psychol Sex Orientat Gend Divers. 2013;1(S):3‐26. 10.1037/0033-2909.129.5.674 PMC2072932 12956539

[18] Link BG , Phelan JC . Conceptualizing stigma. Annu Rev Clin Psychol. 2001;27:363‐385.

[19] Hatzenbuehler ML , Pachankis JE . Stigma and minority stress as social determinants of health among lesbian, gay, bisexual, and transgender youth: research evidence and clinical implications. Pediatr Clin North Am. 2016;63:985‐997. 27865340 10.1016/j.pcl.2016.07.003

[20] Gamarel KE , Nelson KM , Stephenson R , Rivera OJS , Chiaramonte D , Miller RL . Anticipated HIV stigma and delays in regular HIV testing behaviors among sexually‐active young gay, bisexual, and other men who have sex with men and transgender women. AIDS Behav. 2018;22(2):522‐530. 29214408 10.1007/s10461-017-2005-1 PMC5820119

[21] Huebner DM , Davis MC , Nemeroff CJ , Aiken LS . The impact of internalized homophobia on HIV preventative interventions. Am J Community Pathol. 2002;30:327‐348. 10.1023/A:1015325303002 12054033

[22] Golub SA . PrEP stigma: implicit and explicit drivers of disparity. Curr HIV/AIDS Rep. 2018;15(2):190‐197. 29460223 10.1007/s11904-018-0385-0 PMC5884731

[23] Klein H , Washington TA . The pre‐exposure prophylaxis (PrEP) stigma scale: preliminary findings from a pilot study. Int Public Health J. 2019;11:185‐195. 32089789 PMC7034943

[24] Dubov A , Galbo Jr, P , Altice FL , Fraenkel L . Stigma and shame experiences by MSM who take PrEP for HIV prevention: a qualitative study. Am J Men's Health. 2018;12(6):1843‐1854. 30160195 10.1177/1557988318797437 PMC6199453

[25] Szymanski DM , Kashubeck‐West S , Meyer J . Internalized heterosexism: a historical and theoretical overview. Couns Psychol. 2008;36(4):510‐524.

[26] Calabrese SK , Underhill K . How stigma surrounding the use of HIV preexposure prophylaxis undermines prevention and pleasure: a call to destigmatize “Truvada Whores”. Am J Public Health. 2015;105:1960‐1964. 26270298 10.2105/AJPH.2015.302816 PMC4566537

[27] Golub SA , Gamarel KE , Surace A . Demographic differences in PrEP‐related stereotypes: implications for implementation. AIDS Behav. 2017;21(5):1229‐1235. 26143247 10.1007/s10461-015-1129-4 PMC4701641

[28] Mimiaga MJ , Case P , Johnson CV , Safren SA , Mayer KH . Preexposure antiretroviral prophylaxis attitudes in high‐risk Boston area men who report having sex with men: limited knowledge and experience but potential for increased utilization after education. J Acquir Immune Defic Syndr. 2009;50(1):77‐83. 19295337 10.1097/QAI.0b013e31818d5a27 PMC2659469

[29] Bunting SR , Saqueton R , Batteson TJ . A guide for designing student‐led, interprofessional community education initiatives about HIV risk and pre‐exposure prophylaxis. MedEdPORTAL. 2019;15:10818. 31139737 10.15766/mep_2374-8265.10818 PMC6507924

[30] Calabrese SK , Earnshaw VA , Krakower DS , et al. A closer look at racism and heterosexism in medical students' clinical decision‐making related to HIV pre‐exposure prophylaxis (PrEP): implications for PrEP education. AIDS Behav. 2018;22(4):1122‐1138. 29151200 10.1007/s10461-017-1979-z PMC5878986

[31] Calabrese SK , Earnshaw VA , Underhill K , et al. Prevention paradox: medical students are less inclined to prescribe HIV pre‐exposure prophylaxis for patients in highest need. J Int AIDS Soc. 2018;21:6. 10.1002/jia2.25147 PMC6016621 29939488

[32] Marcus JL , Katz KA , Krakower DS , Calabrese SK . Risk compensation and clinical decision making—the case of HIV preexposure prophylaxis. N Engl J Med. 2019;380(6):510‐512. 30726699 10.1056/NEJMp1810743 PMC6396306

[33] Hill C , ed. Consensual Qualitative Research: A Practical Resource for Investigating Social Science Phenomena. American Psychological Association; 2012.

[34] Ching SZ , Wong LP , Said MAB , Lim SH . Meta‐synthesis of qualitative research of pre‐exposure prophylaxis (PrEP) adherence among men who have sex with men (MSM). AIDS Educ Prev. 2020;32(5):416‐431. 33112675 10.1521/aeap.2020.32.5.416

[35] Selfridge M , Card KG , Lundgren K , et al. Exploring nurse‐led HIV pre‐exposure prophylaxis in a community health care clinic. Public Health Nurs. 2020;37(6):871‐879. 32996157 10.1111/phn.12813

[36] Mayer KH , Agwu A , Malebranche D . Barriers to the wider use of pre‐exposure prophylaxis in the United States: a narrative review. Adv Ther. 2020;37(5):1778‐1811. 32232664 10.1007/s12325-020-01295-0 PMC7467490

