# Supplementary material for: Qualitative investigation of factors impacting pre‐exposure prophylaxis initiation and adherence in sexual minority men
Source: Health Expect. 2021 Dec 14;25(1):313–21. doi: 10.1111/hex.13382 (PMC8849378; doi:10.1111/hex.13382)
Supplement: Supplementary file 1 — Supporting information. [file HEX-25-313-s001.docx]

­­­

**Indications (USPSTF)**

- **Men who have sex with men who have 1 of the following characteristics:**
  - A serodiscordant sex partner (partner is HIV+)
  - Inconsistent use of condoms during receptive or insertive anal sex
  - STI (syphilis, gonorrhea, or chlamydia) within the past 6 months
- **Heterosexually active women and men who have 1 of the following characteristics:**
  - A serodiscordant sex partner
  - Inconsistent use of condoms during sex with a partner whose HIV status is unknown and who is at high risk (eg, having a sex partner who injects drugs or a man who has sex with men and women)
  - STI (syphilis or gonorrhea) within the past 6 months
- **Persons who inject drugs and have 1 of the following characteristics:**
  - Shared use of drug injection equipment
  - Risk of sexual acquisition of HIV (see above)

**Contraindications**

- HIV+
- **CrCl <30 for Descovy, CrCl <60 for Truvada**
- HIV exposure <72 hours (consider Post-Exposure Prophylaxis)

**Cautions**

- At risk for kidney disease (DM, HTN, consider monthly Cr)
- Acute viral syndrome in last month (take detailed sexual history, consider HIV viral load)
- Osteoporosis or hx non-traumatic fracture
- Pregnancy or breastfeeding (discuss risks/ benefits)
- HBV infection and ALT >2x ULN (consider ID consult)

**Side effects**

- Overall, PrEP is very safe and well tolerated
- Common to have mild GI discomfort for the first few weeks that will resolve with continued adherence
- 1/200 have renal dysfunction (most all reverse when PrEP discontinued)
- 1% average loss of bone mineral density
- Low risk of headache or weight loss

**First appointment**

- Thorough sexual history**,** counsel on safe sex and harm reduction practices, PrEP does not protect against other STIs besides HIV, discuss importance of medication adherence and follow up.
- Time until effectiveness: 7 days for anal sex, 20 days for vaginal sex and IVDU
- **Initial labs:**
  - **HIV Ag/Ab**
  - **Hep B surface Ag, Hep B core Ab, Hep B surface Ab**
  - **Hep C Ab**
  - **CMP**
  - **RPR**
  - **GC/CT (consider 3 sites: urine, throat, rectal based on risk)**
  - **Pregnancy test (if applicable)**
- **Prescriptions**
  - **All patients:**
    - **Truvada (emtricitabine 200mg/ tenofovir disoproxil 300mg) 1 tab PO daily** (Note: Truvada often preferred by insurance unless indication to start Descovy – current or at risk for renal disease/ osteoporosis)
  - **Cisgender men/ transgender women:**
    - **Descovy (emtricitabine 200mg/ tenofovir alafenamide 25mg) 1 tab PO daily**
- Send to pharmacy after HIV negative, CrCl >30 (Descovy), CrCl >60 (Truvada)
- If concern for Hep B (+s Ag or c Ab) or Hep C infections (+Ab), consider ID consult

**Follow up appointments**

- Initial follow up appointments 3 months after starting PrEP
- **Follow up labs:**
  - **Every 3 months: HIV Ag/Ab, RPR, GC/CT, BMP**
  - Can space BMP to q6 months after first 3 month labs if normal and no risk factors for kidney disease
  - Recommend annual Hep C Ab and Hep B surface Ag (if not immune)
- Continued counseling adherence, safe sex and harm reduction practices
- Remember to vaccinate against HPV and Hep A and B, if indicated

**Additional Resources**

- PrEP hotline, 855-448-7737, 11 am – 8pm EST, Monday - Friday
- PEP hotline, 888-488-4911, 9 am - 9 pm EST, 7 days/ week
